# Reduction of parasitic reaction in high-temperature AlN growth by jet stream gas flow metal–organic vapor phase epitaxy

**DOI:** 10.1038/s41598-022-10937-y

**Published:** 2022-05-10

**Authors:** Kentaro Nagamatsu, Shota Tsuda, Takumi Miyagawa, Reiya Aono, Hideki Hirayama, Yuusuke Takashima, Yoshiki Naoi

**Affiliations:** 1 Institute of Post-LED Photonics, Tokushima University, 2-1 Minami-josanjima, Tokushima, 770-8506 Japan; 2 Graduate School of Advanced Technology and Science, Tokushima University, 2-1 Minami-josanjima, Tokushima, 770-8506 Japan; 3 RIKEN, Institute of Physical and Chemical Research, Wako, 351-0198 Japan

**Keywords:** Engineering, Electrical and electronic engineering

## Abstract

AlGaN-based deep ultraviolet light-emitting diodes (LEDs) have a wide range of applications such as medical diagnostics, gas sensing, and water sterilization. Metal–organic vapor phase epitaxy (MOVPE) method is used for the growth of all-in-one structures, including doped layer and thin multilayers, using metal–organic and gas source raw materials for semiconductor devices. For AlN growth with high crystalline quality, high temperature is necessary to promote the surface migration of Al atoms and Al-free radicals. However, increase in temperature generates parasitic gas-phase prereactions such as adduct formation. In this work, AlN growth at 1500 °C by a stable vapor phase reaction has been achieved by jet stream gas flow metal–organic vapor phase epitaxy. The AlN growth rate increases with gas flow velocity and saturates at ~ 10 m/s at room temperature. Moreover, it is constant at an ammonia flow rate at a V/III ratio from 50 to 220. These results demonstrate the reduction in adduct formation, which is a typical issue with the vapor phase reaction between triethylaluminum and ammonia. The developed method provides the in-plane uniformity of AlN thickness within 5%, a low concentration of unintentionally doped impurities, smooth surface, and decrease in dislocation density because of the suppression of parasitic reactions.

## Introduction

AlGaN-based deep ultraviolet LEDs have a wide range of applications such as medical diagnostics, gas sensing, biochemical agent detection, and water sterilization. Such devices enable the sterilization of home appliances, which are in high demand in the market^1,2^. Recently, deep ultraviolet LEDs have been proposed as a system for reliable sterilization of items from SARS-CoV-2^3–5^. However, a large cooling system for deep ultraviolet LED is required for heat dissipation in known devices because a lot of input power is converted to heat owing to the low efficiency of LEDs^2^. The heat generation is caused by dislocation and point defect nonradiative recombination^6–9^. Metal–organic vapor phase epitaxy (MOVPE) method provides the growth of all-in-one structures, including doped layers and thin multilayers, using metal–organic and gas source raw materials. All semiconductor devices, including LED, LD, and electron devices are manufactured using this method. The growth of the AlN underlying layer for DUV-LEDs is an essential problem for the MOVPE method.

For growing AlN with high crystalline quality, high temperature is necessary to promote the surface migration of Al atoms and Al-free radicals^10–13^.

There are two reaction pathways: an adduct formation by Al and N sources, or a TMAl pyrolysis reaction.

**Figure 1:**
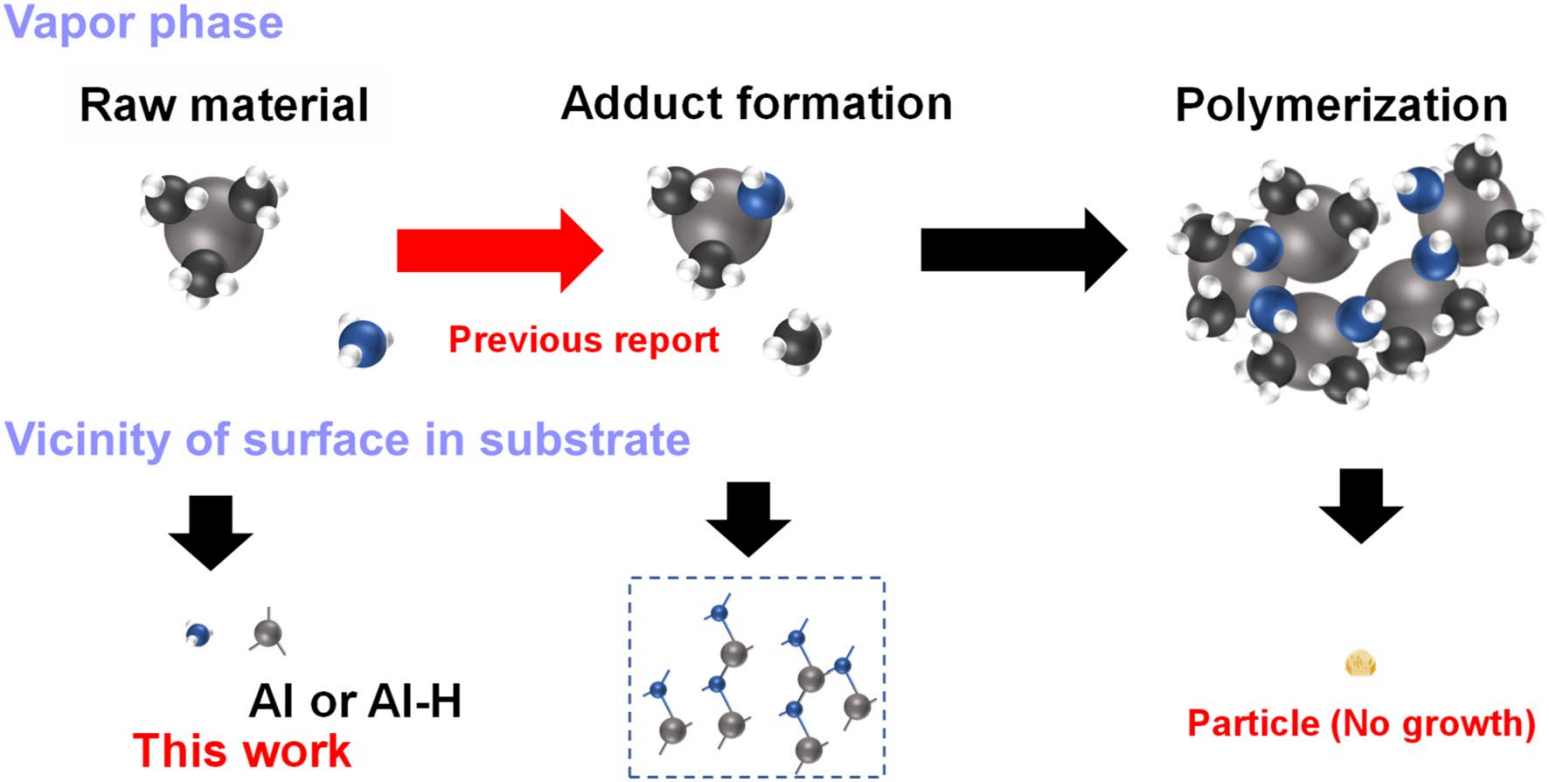
Reaction pathway during raw material transport at AlN growth, and the estimation of final material condition before its deposition on an AlN surface.

However, the increase in temperature generates parasitic gas-phase prereactions such as adduct formation by TMAl and ammonia, as shown in Fig. 1. The parasitic reactions not only cause waste of source gas but also seriously affect the level of unintentionally doped impurities, growth uniformity, and generation of dislocations^10–13^.

Figure 1 shows the schematic representation of raw material transport in a growth reactor. The supplied TMAl raw material forms Al or Al–H at the substrate surface by a pyrolysis reaction, as shown in Eq. (1) and Al–N adduct by parasitic reaction or contaminant particles by polymerization, as shown by Eq. (2).

1 $$TMAl\to DMAl\to MMAl\to Al \,\,or \,\, AlH$$

2 $$TMAl\to TMA{:} \, N{H}_{3}\to {(DMA:N{H}_{3})}_{X}$$

As per a previous report, the adduct formation is unavoidable at TMAl reaction with NH_3_ during the HT-MOCVD process^10,11^. A state of Al–N chemical compound, which is a typical source for AlN growth, is not ideal. The products of nitrogen prereaction with Al–N obstruct the surface migration of Al atoms. Al or Al–H formed by the pyrolysis reaction without the reaction of ammonia possesses a large migration range on the substrate surface^14^. However, adduct polymerization depletes the source materials because particle contamination prevents crystal growth. This problem may be resolved by an alternative supply method from aluminum and nitrogen sources, as reported by Adivarahan et al. and in other works^15–18^. This method not only suppresses the parasitic reaction but also enhances Al migration over the substrate surface; however, it is difficult to increase the growth rate in principle.

Similarly, a growth method with a low NH_3_ flow rate was reported with the suppressed parasitic reaction^19–21^. This technique enabled maintaining the growth rate by reducing NH_3_ supply despite the high temperature. However, metallic Al is deposited at an NH_3_ flow rate below a minimum value. Therefore, the parasitic reaction was strongly suppressed; however, the process was difficult to control in a wider temperature range, e.g., at a temperature of > 1500 °C. Thus, these parasitic reactions in the vapor phase unavoidably occur during AlN growth at high temperatures and high growth rates^10–13^. Therefore, the growth method had to be optimized.

In this study, parasitic reactions were eliminated in the designed and manufactured MOVPE reactor with a high-speed gas flow where gas turbulence was avoided using a mechanism of the combustion chamber in a jet engine. Hence, AlN crystal growth at a high temperature without the parasitic reaction was provided with a low concentration of impurities and a smooth surface.

## Results and discussion

**Figure 2:**
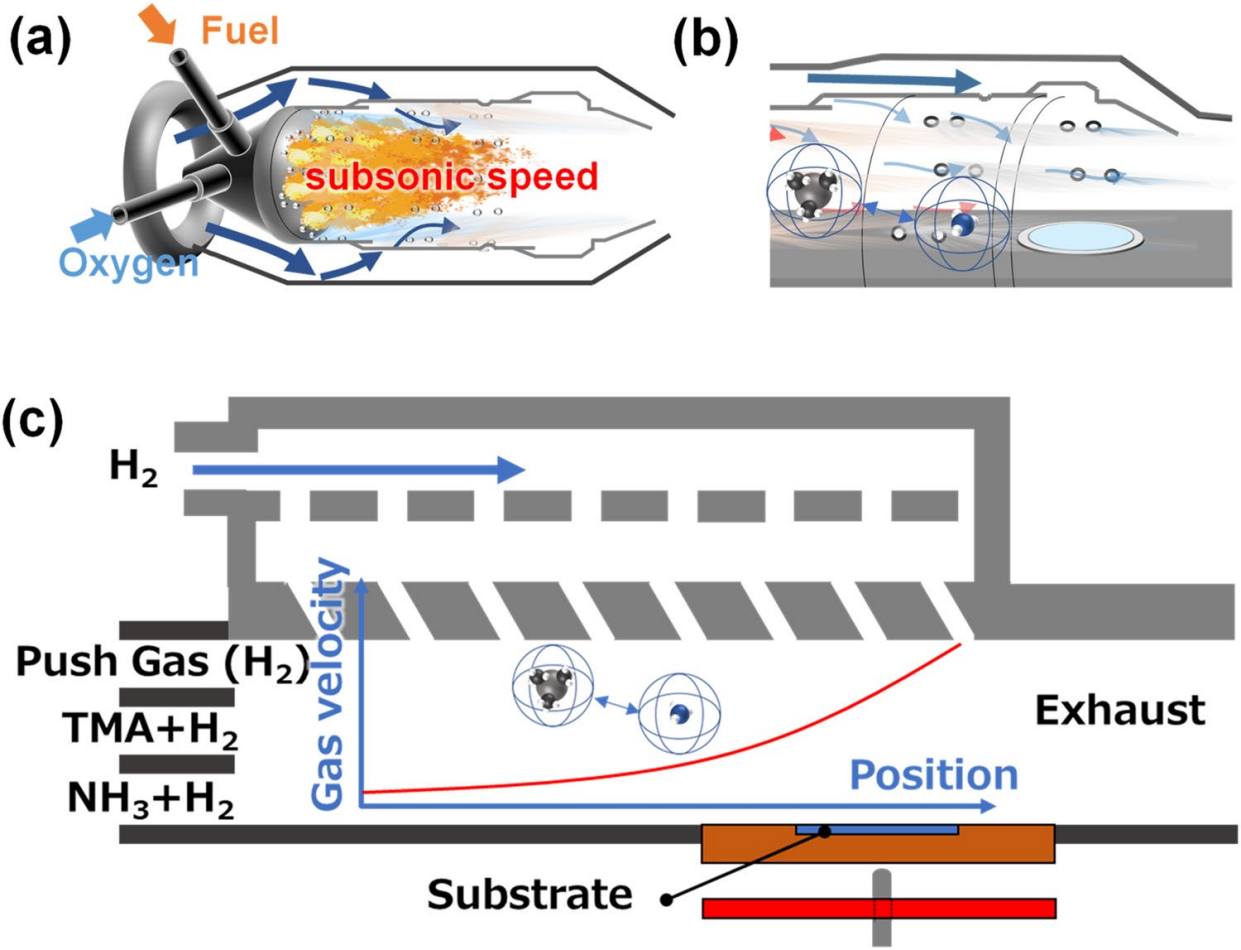
Schematic representation of the growth reactor involving gas flow from the jet engine. (**a**) The heating area in the combustion chamber of a jet engine. The temperature in this chamber exceeds 2000 °C (**b**) An image of raw materials gas flow in the combustion chamber. (**c**) The metal–organic vapor phase epitaxy reactor developed in this study, mimicking a combustion chamber in a jet engine.

A jet engine operating system continuously performs the gas intake, compression, combustion, and exhaustion from the inlet., i.e., a combustion reactor provides a high-temperature laminar gas flow with a high velocity. A combustion chamber comprises a combustion part in the center where fuel burns by mixing with compressed oxygen, whereas the outside bulk is separated by a wall composed of Ti alloy with perforations that provide compressed air inflow and prevent the wall from overheating (Fig. 2a).

Consequently, the chamber wall can be composed of Ti alloy with a lower melting point compared to the temperature in the combustion part heated at > 2000 °C. Moreover, the compressive air is supplied with a controllable speed to the chamber through holes in this wall.

A horizontal-type MOVPE usually uses two or three multilayer flow channels as the gas inlet. The velocity of supply gas flow is determined by the channel cross-section, temperature, and pressure. Hydrogen gas flow from the inlet to the substrate surface (upper-side flow) was provided in our MOVPE reactor by analogy to a jet engine combustion chamber, as shown in Fig. 2b. Figure 2c shows a schematic representation of the MOVPE reactor used in this study. The gas flow increases step by step in the reactor at the same rate as that of the jet gas flow. Tantalum carbide-coated carbon was used as the material of the reactor with an increase in high temperature for safety. The gas flow in conventional MOVPE occurred through three inlet at the maximum on the left-hand side. Therefore, there is one more factor determining the gas flow velocity in this reactor. The MOVPE reactor should be kept clean. Hydrogen gas was selected for the upper-side flow. Moreover, the upper-side gas flow system provides additional options in the selection of heat-resistant materials for the upside flow channel.

**Figure 3:**
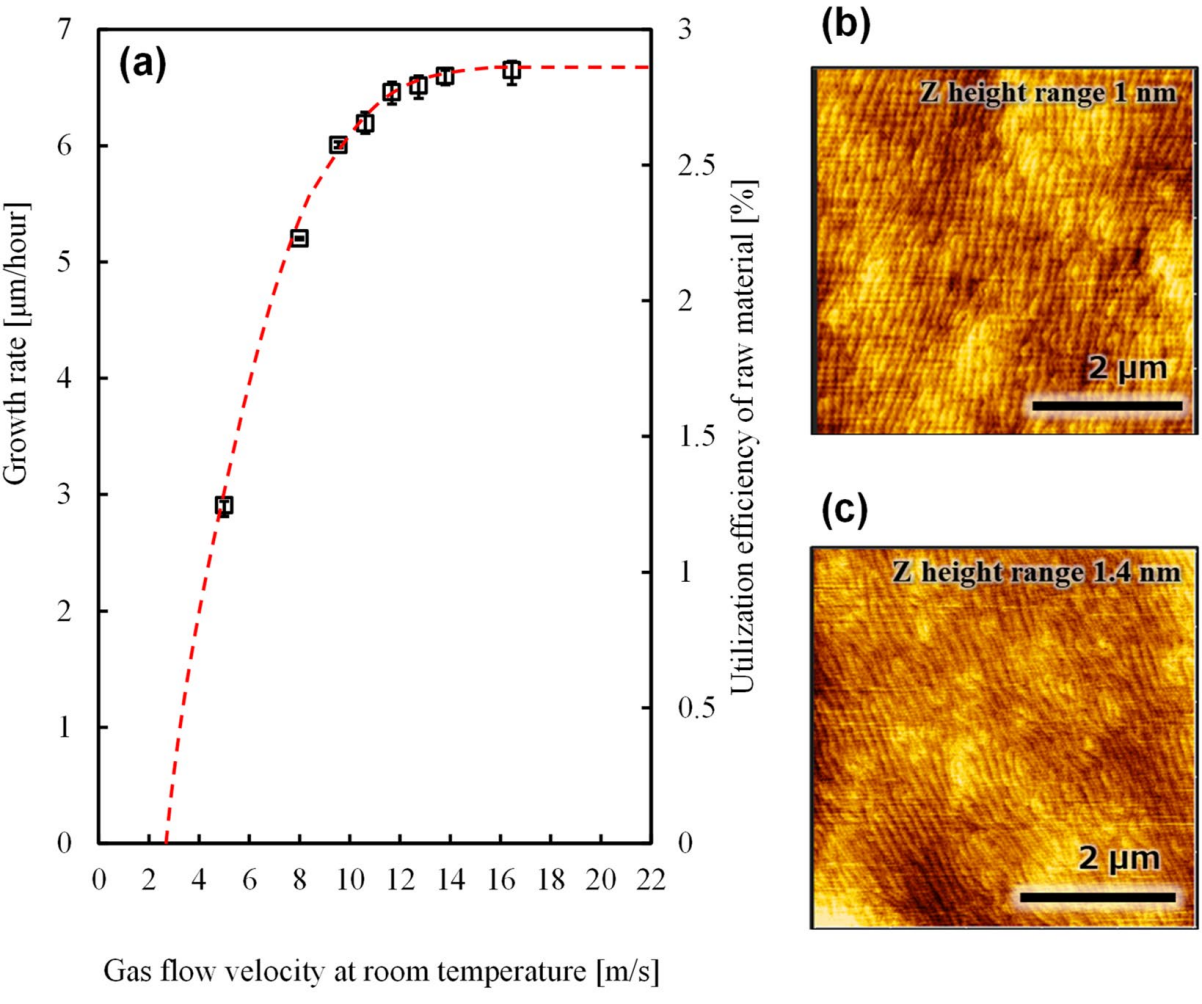
Effect of gas flow velocity. (**a**) The growth rate of AlN layer as a function of gas velocity calculated at room temperature. The adduct formation and polymerization decreased the amount of raw material used for the growth at flow rates below 10 m/s; (**b**) Atomic force microscope (AFM) images of AlN film grown at a gas flow velocity of 10 m/s. The growth rate in the experiment was approximately 20% lower under these conditions compared to the values presented in (**a**); (**c**) AFM images of AlN surface grown at a gas flow velocity of 16.5 m/s.

Figure 3a shows the growth rate at 1500 °C as a function of gas flow velocity at room temperature. The total gas flow velocity increases owing to the reinforcement of the upper-side gas flow. The growth rate increased with total gas flow velocity and saturated at ~ 10 m/s. These results show that the parasitic reaction of the supply material occurs in a weak gas flow. The in-plane thickness uniformity was within 5%. In particular, the material source depletes to < 50% at a gas flow of < 5 m/s, and the dominant type of the supplied raw material are particles that do not contribute to growth. The gas flow velocity in a conventional MOVPE should be 1–2 m/s to provide nonturbulent flow and maintain the substrate^22,23^. Clearly, a high-temperature growth, e.g., at 1500 °C, is complicated because a higher gas flow velocity is necessary. The discussed gas velocities are indicated at room temperature, while actual gas flow velocity at high temperatures should be higher. For example, 15 m/s at room temperature corresponds to 101 m/s on the substrate upside surface at 1500 °C as per our calculations. The quantity of growth material in the microspace increased to provide the reaction without adduct formation in the vapor phase.

The adduct formation by the parasitic reaction is theoretically unavoidable in the case of simultaneous supply with TMAl and NH_3_^10–13^. In other words, the reaction is determined by the probability of the contact between Al and N. Moreover, the growth rate is affected by the parasitic reaction^19,20^.

The obtained surface morphology was afected by the parasitic reaction. Under the condition of 20% loss of growth material with the gas flow velocity of 10 m/s, the atomic step by sapphire disorientation angle is clearly observed using an atomic force microscope in Fig. 3c. However, the surface has macrowaves, curved atomic steps, and microconvexity in the center in contrast to the image taken at 16.5 m/s (Fig. 3b). Moreover, the terrace length uniformity was clearly improved (Fig. 3b,c and 3c) with the reduction of the parasitic reaction. The X-ray full width at half maximum value for (0002) and (10–12) diffractions of grown AlN layer were 250 and 580 arcsec, respectively, in the sample in Fig. 3c, and 160 and 310 arcsec, respectively, in the sample in Fig. 3b.

**Figure 4:**
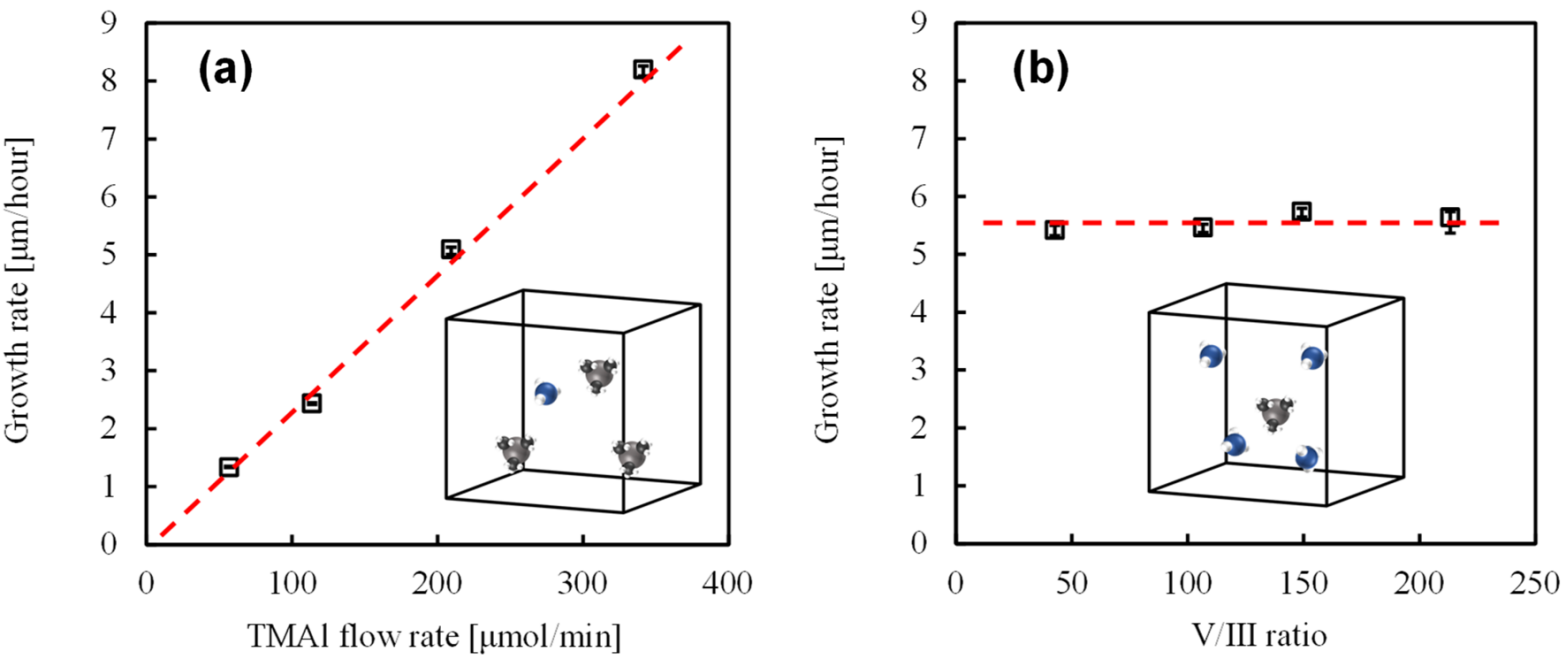
Evidence of suppressed parasitic reaction at an increasing growth rate at a gas flow velocity of 16.5 m/s. (**a**) AlN growth rate vs. TMAl flow rate. Increasing Al source concentration in certain areas result in adduct formation and growth rate saturation; (**b**) Dependence of AlN growth rate on ammonia flow rate. Increasing N source concentration in a certain area increases the adduct formation probability, similar to (**a**). The growth rate decreases with an increasing ammonia flow rate in the case of adduct formation.

The effect of parasitic reaction on the AlN growth rate was examined at different TMAl flow rates (Fig. 4a). AlN growth rate linearly increases up to 8 μm/h with the TMAl flow rate, unlike previous studies^20,21^, where the growth rate saturated at ~ 1 μm/h owing to a parasitic reaction. Hence, the dependence in Fig. 4b shows that the increased NH_3_ flow rate impedes the transportation of Al compound by promoting the parasitic reaction. Therefore, in^19–21^, the growth rate decreased with increasing NH_3_ content. Our results show that the parasitic reaction does not occur at V/III ratios from 50 to 220 (Fig. 4b).

**Figure 5:**
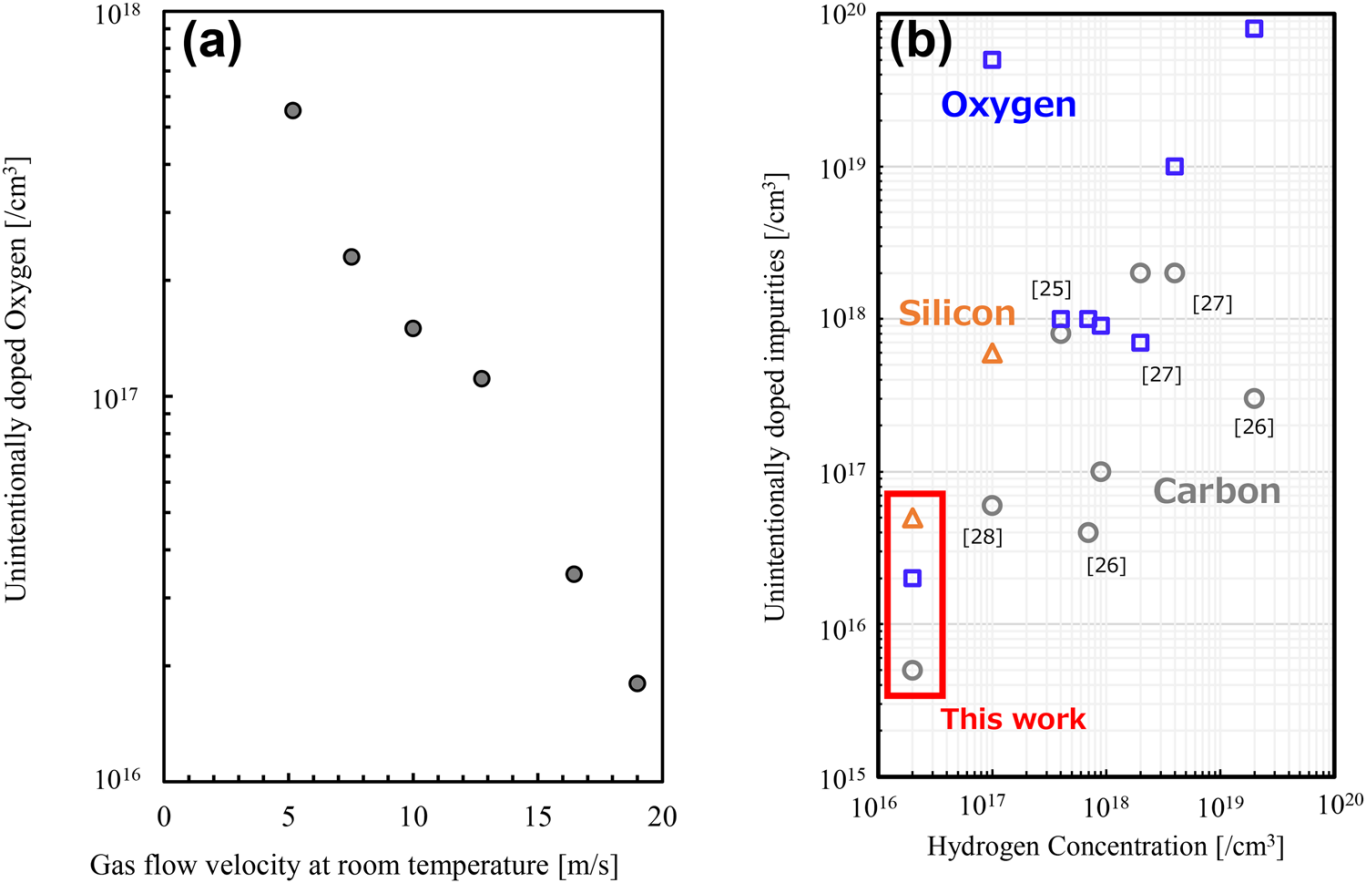
The impurity concentrations ingrown AlN. (**a**) Oxygen admixture concentration in the AlN measured using secondary ion mass spectrometry (SIMS) as the function of gas flow velocity grown under fixed growth temperature of 1500 °C. This figure demonstrates the efficiency of increasing gas flow for the reduction of oxygen concentration. The data were obtained at a depth of around 500 nm below the crystal surface; (**b**) Comparison between the best data impurity concentrations measured by SIMS obtained in this work compared to those reported in the literature^25–28^.

The low concentrations of oxygen, silicon, and carbon impurities in films should be demonstrated to confirm the advantages of the proposed growth process. In particular, the oxygen concentration was reported to affect optical properties by forming oxygen-vacancy complexes; hence, reduction of its concentration is important^24^. Moreover, oxygen is always generated upon sapphire (Al_2_O_3_) heating. The oxygen concentration decreases with an increase in gas flow velocity (Fig. 5a). This phenomenon can be explained by the quick exhaustion of generated oxygen. The data on admixture concentrations in AlN obtained in this work and reported in the literature are demonstrated in Fig. 5b. Importantly, the concentrations of all impurities in AlN grown in this study are lower by how many orders of magnitude compared with those reported in previous studies^25–28^. A high temperature of 1500 °C and a high gas velocity of 16.5 m/s provided such impurity concentrations. The reduction in carbon impurities are similar to that of GaN, and the methyl group of transport materials decompose with an increase in temperature. Then, the carbon impurities reduce because of the decomposed methyl group forming methane^29^.

Moreover, conventional MOVPE system uses quartz at the flow channel. In that case, Si impurities are contaminations from the quartz material that decomposed at > 1200 °C. Our system was cooling the upper side flow-channel as the highest temperature region by the jet stream gas flow. Moreover, our equipment did not contain quartz. Consequently, the concentration of the unintentional Si impurities is considerably less.

To summarize, we designed and manufactured an MOVPE reactor providing stable crystal growth even at high gas flow velocities. The growth rate of AlN on the sapphire substrate increased by increasing the gas flow velocity. The process of AlN growth by MOVPE with a high gas flow velocity effectively reduces the parasitic reaction between TMAl and ammonia. Consequently, a low concentration of unintentionally doped impurities and smooth film surface were obtained because of the suppression of this parasitic reaction. Finally, the reduction in dislocation density was confirmed by XRD.

## Method

AlN layers were grown on the (0001) sapphire substrate using the horizontal type MOVPE with a jet engine gas flow system. The misorientation of the sapphire substrate from m-plane was 0.2°. The reactor was coated with carbonized tantalum to ensure thermal and corrosion resistance. This equipment can withstand temperatures of up to 2000 °C. The growth of AlN was attributed to the continuous flow of TMA carrier gas and ammonia; herein, TMAl and ammonia were used as Al and N precursors, respectively. The growth rate was primarily determined using the TMA flow rate, as the ammonia flow rate was a hundred times faster. Hydrogen was used in the reactor without ammonia as a carrier gas and a heater and reactor purge gas to avoid molecular nitrogen pyrolysis. The growth temperature was simultaneously measured using a thermocouple and dual-wavelength pyrometer to control the pyrometer indications. Firstly, 5-nm thick AlN buffer layers were deposited at 950 °C. Secondly, 650-nm thick first AlN layers were grown at 1500 °C to guarantee stable crystalline quality. Then, the second AlN layers were grown on the first layers. The standard growth process temperature and duration were 1500 °C and 30 min, respectively. The TMA and NH_3_ flow rates were 209 μmol/min and 45 mmol/min, respectively.

The gas flow velocities at room temperature were calculated using the following equation.

3 $$Flow\,\, velocity\left(R.T\right)=\frac{F}{A}\times \frac{{P}_{G}}{101.3kPa}$$

where F is the total flow rate, A is the cross-section area, P_G_ is the growth pressure in the reactor. The gas flow velocities at the growth temperature of 1500 °C were calculated by computational fluid dynamics analysis (simulation).

The atomic layer and surface roughness in AlN were measured by AFM (HITACHI corporation 5500 M). To measure the crystalline quality, X-ray diffraction rocking curve measurement was obtained at Smart Lab (Rigaku Corporation). Moreover, the FWHM of XRD data was confirmed, and the correlation with dislocation densities using a transmission electron microscope provided by the Foundation for Promotion of Material Science and Technology of Japan (MST) was obtained. SIMS measurement was measured by MST, and AlN growth thickness was measured by an optical interference film thickness meter.

## Data Availability

The data that support the findings of this study are available from the corresponding author upon reasonable request.
